# Vedolizumab is superior to infliximab in biologic naïve patients with ulcerative colitis

**DOI:** 10.1038/s41598-023-28907-3

**Published:** 2023-02-01

**Authors:** Renato Sablich, Maria Teresa Urbano, Marco Scarpa, Federico Scognamiglio, Alberto Paviotti, Edoardo Savarino

**Affiliations:** 1 Gastroenterology Unit, Santa Maria Degli Angeli Hospital, Pordenone, Italy; 2 Clinica Chirurgica 3, Azienda Ospedale Università Di Padova, Padua, Italy; 3 Department of Surgery, Oncology and Gastroenterology, University of Padua, Padua, Italy; 4 Gastroenterology Unit, Azienda Ospedale Università Di Padova, Padua, Italy

**Keywords:** Gastrointestinal system, Gastroenterology, Inflammatory bowel disease

## Abstract

There are no prospective, head-to-head, controlled trials comparing the efficacy and safety of Infliximab (IFX) and Vedolizumab (VDZ) for the treatment of moderate-to-severe ulcerative colitis (UC), while only a few real-life retrospective studies have been published so far. We assessed the efficacy of IFX vs. VDZ in two cohorts of biologic-naïve outpatients with moderate-to-severe UC or mild, but refractory, disease. Data were extracted from patients’ files and reviewed. The duration of follow-up (FU) was 52 weeks. The primary endpoint was the clinical remission (CR) at the end of FU. Secondary endpoints were: drug persistency, time to obtain CR, clinical response at the end of the induction phase (IP), steroid-free CR (compared to patients who used steroids at baseline) at the end of FU, need for drug optimization, adverse events (AEs), and normalization of C-reactive protein (CRP). We also analyzed the causes of dropping out (primary non-response), or secondary loss of response (immunogenic or not), for each group. We enrolled 82 patients (50 IFX and 32 VDZ) who met the inclusion criteria. At the end of FU, CR was obtained in 32% of the patients on IFX and 75% on VDZ (p = 0.0003). Drug persistency was superior for VDZ compared to IFX (78% vs. 52%, p = 0.033). Clinical response at the end of induction was reached in 54% and in 81% in the IFX and VDZ group, respectively (p = 0.014). Steroid-free clinical remission at the end of FU was 62% and 94% in the IFX vs. VDZ group, respectively (p = 0.036). The need for drug optimization was higher for VDZ than for IFX (28% vs. 57%, p = 0.009), while the time to obtain CR, the incidence of AEs, mean duration of FU, and rate of CRP normalization at the end of IP were comparable between the two groups. There was a prevalence of patients dropping out because of primary non-response in IFX group (p = 0.027), while the incidence of secondary loss of response was similar in the two groups. At the multivariate analysis, CRP and Partial Mayo Score (PMS) at T0 did not correlate with CR at the end of FU in both groups. In this retrospective, real world data study in biologic-naïve patients, VDZ was superior to IFX in CR, clinical response rate at the end of IP, drug persistency, steroid-free remission, and need for optimization at the end of FU.

## Introduction

Ulcerative colitis (UC) is a chronic relapsing and remitting inflammatory disease, involving different extensions of the colonic mucosa, causing disabling and sometimes life-threatening conditions^1^, need for continuous medical treatment, and surgery in a substantial proportion of patients^2,3^. The incidence is relatively stable in Western Countries, but rapidly increasing in developing areas. The estimated incidence in Italy is 12.8/100,000/year for females and 15.8/100,000/year for males^4^ with a prevalence of about 190/100,000^5^. Etiology is attributed to an abnormal immunologic response to luminal intestinal antigens in genetically predisposed subjects.

Conventional medical therapy relies on 5-ASA, corticosteroids, and thiopurines^6^, but a considerable proportion of patients with a moderate-to-severe disease needs biologics due to refractoriness, intolerance, or steroid dependency^7,8^. Two classes of biologics are currently approved in Italy for UC. Anti-Tumor Necrosis Factor alfa (anti-TNFα) class includes infliximab (IFX), a mouse-derived chimeric monoclonal antibody, and adalimumab (ADA) or golimumab (GOL) that are human cell-derived^9^. The mechanism of action is expressed by binding the soluble and transmembrane forms of TNF-α, a pro-inflammatory cytokine secreted by macrophages, monocytes, and T lymphocytes that plays a pivotal role in activating the inflammatory cascade^10^. The class of anti-integrin monoclonal antibodies, which includes vedolizumab (VDZ), selectively binds the α4β7 integrin on the surface of circulating lymphocytes, preventing their interaction with the adhesion MAdCAM-1 receptor on the endothelial cells of intestinal vasculature, down regulating the trans-endothelial leukocyte trafficking, and reducing the recruitment of inflammatory cells across the mucosal layers^11^. Both IFX and VDZ are administered intravenously and were approved by the Italian regulatory agency in 2006 and 2014 respectively as first-line treatment in UC after the failure of conventional therapy^12^.

Biologics have clearly offered a new perspective in the management of Inflammatory Bowel Diseases (IBD). In the ACT1 phase 3 randomized, double-blind, placebo-controlled trial, 69.4% of patients with UC refractory to conventional therapy responded to IFX at week 8, and 45.5% maintained the response at week 54^13^. In the GEMINI 1 double blind, placebo-controlled, randomized, phase 3 trial, the response rate for VDZ at week 6 was 47.1%, and the remission rate at week 52 for VDZ every 8 weeks was 41.8%^14^. An indirect comparison between ACT and GEMINI trials is, however, improper since the two studies had a different design and enrolled a different IBD population, with 48% of subjects previously exposed to anti-TNFα in the GEMINI trial.

Nevertheless, despite the substantial rate of response and remission, about 40% of the patients experience a primary non-response to IFX and a secondary loss of response due to various mechanisms, including the development of neutralizing antibodies in 30–40% of cases^15^. Furthermore, an increased rate of opportunistic infections, severe infections, and malignancies has been associated with the use of IFX^16^. Conversely, VDZ has a negligible risk of developing neutralizing antibodies and the risk for infections is generally deemed lower than that of IFX. Furthermore, the risk of cancer, although extremely low for IFX, seems to be even null for VDZ^17^. However, the majority of the latter data come from retrospective studies enrolling patients with previous exposure to different treatments, including anti-TNFα, while real-life comparative studies in biologic naive patients are limited.

We conducted a retrospective, real world, single-center study with a 52-week follow-up (FU), to compare the efficacy of IFX and VDZ in two almost homogeneous cohorts of biologic-naïve patients with moderate-to-severe UC in terms of clinical response, rate of clinical remission (CR), time to CR, steroid-free remission, need for optimization, drug persistency, normalization of C-reactive protein (CRP), and safety.

## Materials and methods

### Study design, patient population and selection criteria

This retrospective, real world data study included consecutive adult outpatients with refractory UC unexposed to biologics or small molecules, enrolled in the IBD Unit of Pordenone Hospital (Pordenone, Italy), between April 2015 and September 2020 for IFX, and between December 2016 and October 2020 for VDZ. IFX and VDZ were administered intravenously (IV) according to standard induction protocol at 0, 2, and 6 weeks, followed by maintenance every 8 weeks. IFX was used at the dose of 5 mg/kg and VDZ at the dose of 300 mg. The choice of the biologic was guided primarily by physicians' personal confidence and belief about the efficacy and safety of the two agents.

The study was performed by using clinical, laboratory, and endoscopic data collected in the medical registry of our institution. Patients were selected during routine clinical follow-up using the data already stored in the medical records and were not exposed to any additional intervention for research purposes. Therefore, according to the current Italian regulations, submission to and approval by the Regional Ethical Committee were not required. All participants provided written informed consent for the use of their medical data in an aggregate and anonymous form.

The disease activity was assessed on a clinical basis according with the Partial Mayo Score (PMS) and on endoscopic basis according to the Endoscopic Mayo Score. Inclusion criteria were: age ≥ 18 years, diagnosis of UC with moderate-to-severe activity according to Clinical Mayo Score (PMS ≥ 5), mild UC according to PMS (PMS < 5) but refractoriness to conventional immunosuppressive drugs, and endoscopic Mayo score ≥ 2. Exclusion criteria were: the absence of written consent to the use of anonymous personal data for research purpose, previous exposure to any biologic, diagnosis of indeterminate colitis, presence of symptomatic colonic stenosis, presence or past history of malignancy in the last 5 years, and pregnancy for both groups. Furthermore, in the IFX group we excluded patients in whom IFX was used as rescue therapy in hospitalized critical patients with acute severe UC unresponsive to a five-day IV steroids regimen.

PMS was assessed before the induction (T0) and before each infusion. Disease activity was defined as mild with a PMS between 2 and 4, moderate with a PMS between 5 and 7, and severe if the PMS was ≥ 7. CR was defined as a PMS equal to 0 or 1 with a bleeding sub-score of 0. Endoscopic activity was assessed by Endoscopic Mayo score at baseline. Other covariates analyzed at baseline were: mean age at diagnosis, gender, disease extension and duration (years from diagnosis to enrollment), CRP, albumin level, use of aminosalicylates (5-ASA), corticosteroids (CCS), thiopurines or methotrexate (MTX) at baseline and continued for at least 4 weeks during induction or combination therapy with thiopurines continued for 6 months (Table 1). CRP was expressed as mg/dL (normal range 0.0 to 0.5) and albumin as g/dl (normal range 3.5 to 5.5).

**Table 1:** Clinical characteristics of the study populations.

	IFX (n = 50)	VDZ (n = 32)	p value
Mean age of diagnosis (± STD)	35.4 (± 13.8)	47.2 (± 17.8)	**0.001**
Male, n (%), female, n (%)	33 (66%); 17 (34%)	17 (53%); 15 (47%)	0.448
UC extension, n (%)	Proctitis, 3 (6%)	Proctitis, 0 (0%)	0.259
	Left sided, 21 (42%)	Left sided, 17 (53%)	0.260
	Extended, 26 (52%)	Extended, 15 (47%)	0.650
Duration of disease in years, mean ± STD	9.5 (± 9.29)	8 (± 8.56)	0.473
PMS at T0, mean ± STD	5.32 (± 1.96)	4.84 (± 2.03)	0.289
Mild UC (PMS 2–4), n (%)	15 (30%)	10 (31%)	0.904
Moderate UC (PMS 5–7), n (%)	30 (60%)	19 (60%)	0.955
Severe UC (PMS > 7), n (%)	5 (10%)	3 (9%)	0.925
CRP, mean ± STD	1.42 (± 2.29)	1.9 (± 2.57)	0.380
Albumin value, mean ± STD	3.76 ± 0.58	3.54 ± 0.77	0.078
5-ASA use, n (%)	22 (44%)	14 (43%)	0.982
Steroid use, n (%)	21 (42%)	18 (56%)	0.750
Thiopurine use, n (%)	11 (22%)	6 (19%)	0.723
COMBO with thiopurine for 6 months, n (%)	9 (18%)	5 (16%)	0.463
MTX use, n (%)	1 (2%)	1 (3%)	0.625

p value was obtained by chi-square, exact Fisher, and *t* Student’s test.

*STD* standard deviation (STD), *PMS* partial mayo score, *CRP* C-reactive protein, *MTX* Methotrexate.

Significant values are in bold.

### Data collection and variable definitions

Data were extracted from the IBD-dedicated electronic database in use at our Unit. We controlled for the following variables at T0 to select study groups: gender, disease duration and extension, mean PMS at T0 to define mild, moderate and severe UC, endoscopic activity defined by the Mayo Endoscopic Score as moderate or severe (Mayo score ≥ 2), CRP at T0, albumin at T0, oral or topical steroids, thiopurines, MTX e 5-ASA in use at T0 and up to 4 weeks beyond, and combination therapy with thiopurines continued for at least 6 months.

### Follow-up

The end of the FU was set at week 52. The mean follow up was calculated by including patients who discontinued treatment prior to week 52 due to primary non-response, secondary loss of response, AEs, or the need for surgery. The PMS was calculated at each infusion. In the case of non-response or worsening of clinical condition, drug trough levels were measured, and endoscopic activity reassessed by recto-sigmoidoscopy when considered appropriate. Steroids or immunosuppressants were added when deemed necessary by the physician during the FU. The need for dose optimization or drug switch was also reported.

### Definition of outcomes

The primary endpoint was the CR at the end of FU. Secondary endpoints were the mean duration of FU, drug persistency, time to obtain CR, clinical response at the end of induction (which was set for both the groups at 14 weeks), steroid-free CR at the end of FU (compared to patients who used steroids at baseline), need for drug optimization, CRP normalization, and adverse events (AEs). CR was defined as PMS equal to 0 or 1 with subscore 0 for bleeding. Persistence in therapy was defined as non-interruption of the drug within the end of FU. Clinical response was defined as decrease of at least 2 points in PMS. Steroid-free remission was defined as no need for systemic or topical steroids during the observation period (that is no steroid therapy when remission was achieved). The need for optimization indicated a change in the dosage of the drug, or in the interval between infusions: for IFX, dose optimization was intended as dose escalation to 10 mg/kg in patients induced with 5 mg/Kg or shortening of the interval to 4 weeks regardless of the initial dose; while for VDZ, only shortening of the interval at 4 weeks without dose adjustment was allowed. CRP normalization indicated a value in the range of normality (< 0.5 mg/dl). The occurrence of any new symptom or clinical condition was considered an AE. We also analyzed the causes of dropping out before the end of the FU, distinguishing between primary non-response (absence of clinical improvement in the first 4–6 weeks since the beginning of the drug), or secondary loss of response (clinical worsening after initial response).

### Statistical analysis

Since, by their nature, observational studies make results vulnerable to confounders, major covariates were compared at baseline before and after propensity score weighting (Tables 1, 2). Since patients were not randomly assigned to IFX or VDZ group, a two-arm propensity score weighted analysis was performed at baseline to reduce the effect of selection bias and simulate randomization. The conditional probabilities of receiving IFX or VDZ treatment given the observed covariates were estimated using a nearest neighbour matching model algorithm. This model included the following variables: age of diagnosis, extension and duration of disease, PMS, albumin value and CRP at T0. Overlap of the propensity score distributions was assessed examining a graph of propensity scores across treatments^22^. Sample homogeneity was assessed by Student’s *t* test for continuous variables reported as mean and standard deviation (STD), while chi-square test and exact Fisher test were used for categorical ones expressed as frequencies and percentages. P-value < 0.05 was considered statistically significant. In the overall population and in each treatment group, the univariate logistic regression analysis was applied to eligible predictive factors like CR, clinical response, steroid-free remission, need for optimization, and safety. Results were presented as odds ratio (OR) with 95% confidence intervals (95% CI). Treatment persistence and time to obtain CR were respectively measured from inclusion up to the drug withdrawal and from the inclusion up to the achieved remission, by Weighted Kaplan–Meier analyses with 95% CI, and log-rank tests were used to compare Weighted Kaplan–Meier rates. The normalization of CRP was expressed as OR. The multivariate logistic regression analysis was applied to compare the effect of CRP and PMS at baseline on the clinical remission at the end of FU. All analyses were conducted using IBM SPSS (Statistical Package for Social Science) version 26.0 software.

**Table 2:** Clinical characteristics of the study populations after propensity score weighting.

	IFX			VDZ			*T* test
	Median	Low quartile	Upper quartile	Median	Low quartile	Upper quartile	p value
Age of diagnosis	46	39	52	37	32	55	0.75
UC extension	3	2	3	3	2	3	0.60
Duration of disease	7	2	11	5	2	10	0.76
PMS at T0	5	3	7	5	3	7	0.76
Albumin value	3.6	3.25	4.05	3.9	3.425	4	0.62
CRP at T0	0.8	0.55	1.2	0.75	0.3	2.3075	0.12

## Results

According to the inclusion and exclusion criteria, we included 100 biologic-naïve patients, 68 on IFX, and 32 on VDZ. In the IFX group, seventeen patients with severe acute UC required rescue therapy with IFX after failure of IV steroids and were excluded from the analysis. One additional patient was excluded because of the uncertain classification of his colitis.

**Figure 1:**
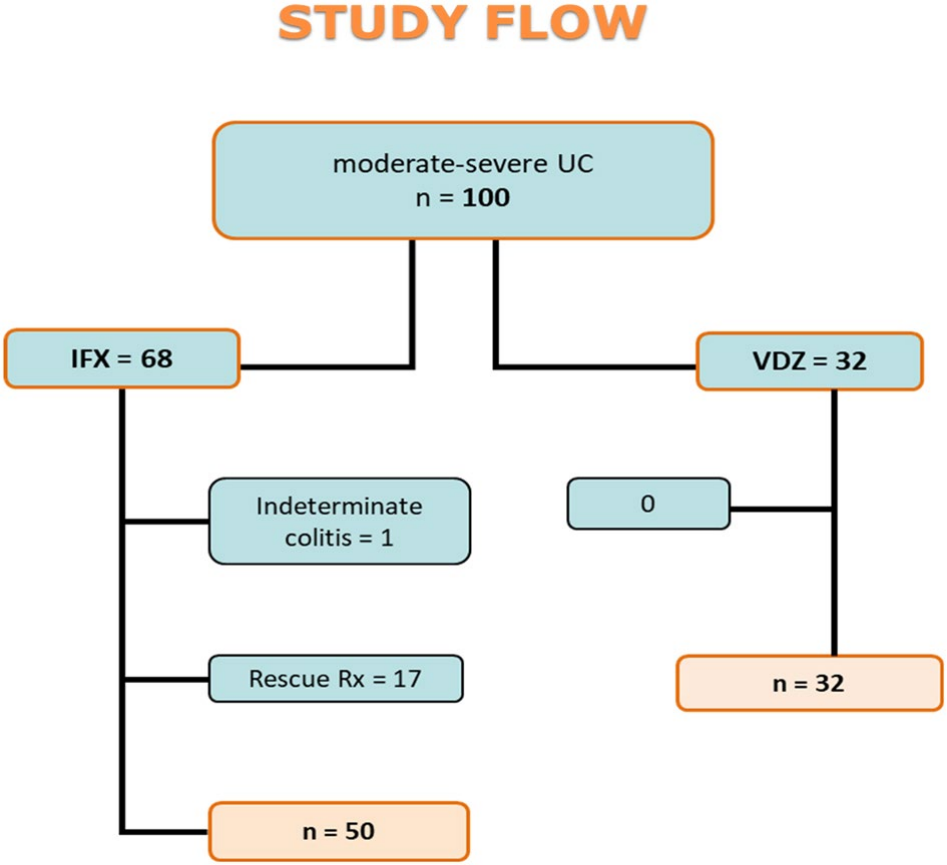
Study flow chart.

All the VDZ patients were eligible. Eighty-two patients were finally enrolled in the study (Fig. 1). In the two cohorts, CRP was available in 47 and 30 patients, and albumin in 42 and 23 in the IFX and VDZ group respectively. Plots of standardized mean differences at baseline, before and after propensity score weighting, are shown in Tables 1 and 2, respectively. The two groups were homogeneous for the following covariates: gender, disease extension, duration of illness, severity at the beginning of biological therapy (mean PMS and its sub-scores, endoscopic activity, CRP, and albumin at baseline), and drugs in use at T0. Before application of propensity score (but not confirmed after its application): the mean age at diagnosis was higher in the VDZ than in the IFX group (mean age 47.2 vs. 35.4, p = 0.001), but this was not confirmed after propensity score analysis. Thus, the two groups showed an adequate overlapping of baseline characteristics for all analysed covariates.

Not all the patients completed the observation period of 52 weeks. Indeed, 25 patients on VDZ and 27 on IFX completed the 52-week FU, but the mean FU was homogeneous in the two groups (37 weeks for IFX group vs. 45 weeks for VDZ; p = 0.154).

**Figure 2:**
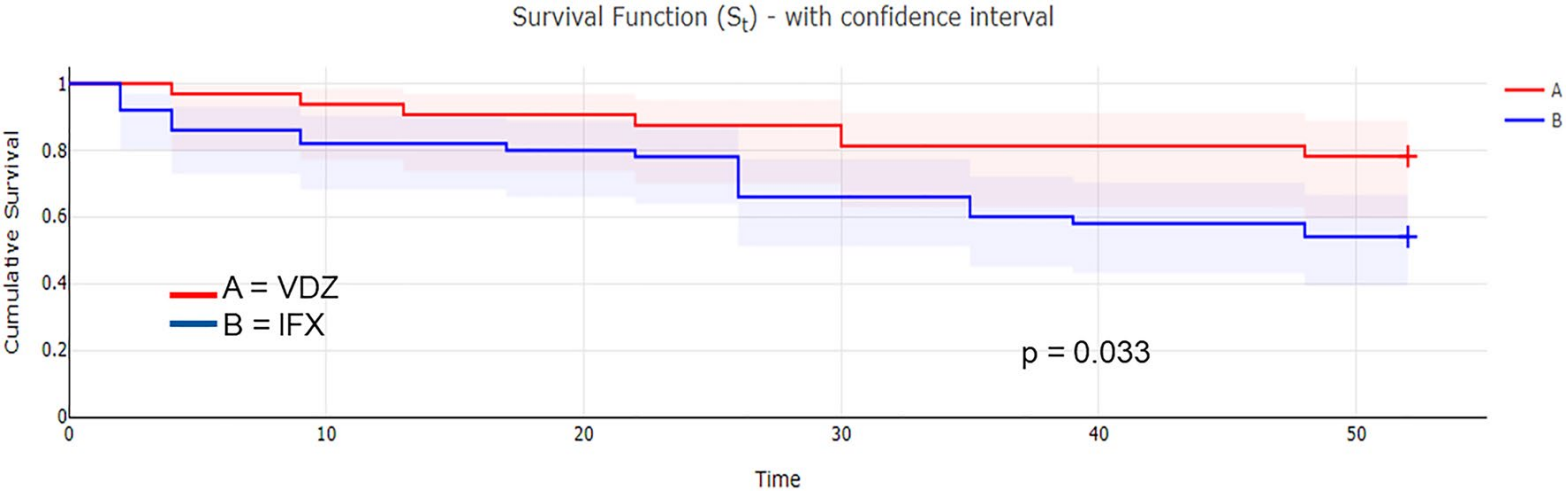
Drug persistency of Infliximab (IFX) and Vedolizumab (VDZ) according to Weighted Kaplan–Meier analysis at 52 weeks.

**Figure 3:**
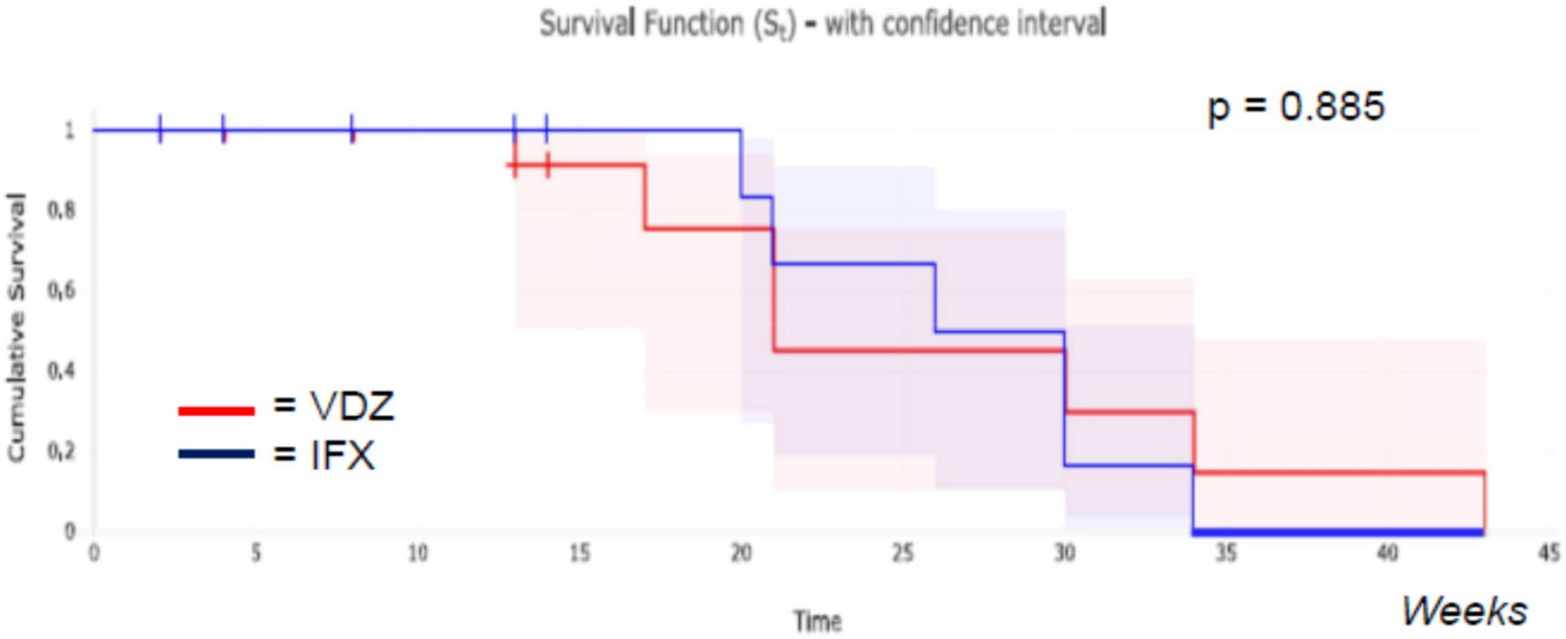
Time to obtain clinical remission (CR) in Infliximab (IFX) and Vedolizumab (VDZ) group, according to Weighted Kaplan–Meier analysis.

Results are summarized in Tables 3, 4 and 5. The primary endpoint of CR at the end of FU was achieved by 75% and 32% of the patients taking VDZ and IFX respectively (p = 0.0003; OR: 6.375; 95% CI 2.353–17.251). In the multivariate analysis, the CR at the end of FU was not associated with CRP and PMS at baseline in either group. Among secondary outcomes, drug persistency was higher for VDZ than for IFX (VDZ 78% vs. IFX 52%, p = 0.033) as shown by the Weighted Kaplan–Meier curve (Fig. 2). The response rate was also higher in the VDZ group (81% for VDZ vs. 54% for IFX, p = 0.014; OR: 3.69; 95% CI 1.29–10.52). The steroid-free remission at the end of FU was observed in 94% of the subjects on VDZ and in 62% of those on IFX with a statistically significant difference (p = 0.036; OR: 10.461; CI 1.158–94.48). The median time to achieve CR (p = 0.88) was not statistically different, as shown by the Weighted Kaplan–Meier curve (Fig. 3). Also, the mean FU (p = 0.154) was similar between the two groups, while the need for optimization was less for VDZ than for IFX (28% vs. 57%, p = 0.009; OR: 3.53; 95% CI 1.36–9.15).

**Table 3:** Main results obtained comparing Infliximab (IFX) and Vedolizumab (VDZ) in naïve patients with Ulcerative Colitis.

	IFX (n = 50)	VDZ (n = 32)	p value
Duration of clinical FU, mean (weeks)	37	45	0.154
Subjects in CR at the end of FU, n (%)	16 (32%)	24 (75%)	**0.0003**
Drug persistency at the end of FU, n (%)	26 (52%)	25 (78%)	**0.033**
Subjects who achieved CR at the end of FU	23	21	0.130
Time to obtain CR (weeks, median)	8.7	13	0.885
Response at the end of induction, n (%)	27 (54%)	26 (81%)	**0.014**
Steroid-free remission at the end of FU compared to patients who used steroids at baseline, n (%)	13/21 (62%)	17/18 (94%)	**0.036**
Need for optimization, n (%)	29 (57%)	9 (28%)	**0.009**
Primary non-response	15 (30%)	3 (9%)	**0.027**
Secondary loss of response (immunogenic or not)	3 (6%)	3 (9%)	0.327
Adverse events, n (%)	7 (14%)	1 (3%)	0.139
CRP normalization at the end of induction, n (%)	15/38 (39%)	10/19 (53%)	0.347

p value was obtained by chi-square, exact Fisher, odds ratio, log-rank test, and *t* Student’s test.

*FU* follow-up, *CRP* C-reactive protein.

Significant values are in bold.

**Table 4:** Multivariable logistic regression for CRP and PMS at T0 in the IFX group (n = 15).

	95% CI	p value
Remission at the end of FU combined with CRP at T0 and PMS at T0	− 11.42 to 11.33	0.993

Type: independent values (CRP and PMS at T0).

*CI* confidence interval.

**Table 5:** Multivariable logistic regression for CRP and PMS at T0 in the VDZ group (n = 19).

	95% CI	p value
Remission at the end of FU combined with CRP at T0 and PMS at T0	− 5.72 to 30.56	0.167

Type: independent values (CRP and PMS at T0).

*CI* confidence interval.

We compared the normalization rate of CRP in the two groups at the end of induction phase (IP). CRP at baseline and at the end of IP was available in 38 patients for IFX and 19 for VDZ. Normalization occurred in 15 IFX and 10 VDZ patients without statistically significant difference (p = 0.347; OR: 1.7; 95% CI 0.56–5.17).

AEs occurred in one patient on VDZ (arthritic syndrome) and in seven on IFX. In detail: psoriasis (n = 1), abdominal tuberculosis (n = 1), palpitation (n = 1), skin reaction (n = 3), and muscle aches (n = 1). Although higher in the IFX group (14% vs. 3%), the difference was not statistically significant (p = 0.139; OR: 5.04; 95% CI 0.59–43.13).

The percentage of primary non-response was higher in IFX group (30%) vs. VDZ (9%), with a significant difference (p = 0.027) between the two groups. Secondary loss of response was similar (p = 0.327).

## Discussion

Few studies have compared anti-TNF and anti-integrins in biologic-naïve patients with UC so far. The aim of the present one was to compare the efficacy and safety of two intravenous biologics (IFX and VDZ), with a different mechanism of action, both approved as first-line treatment for moderate-to-severe UC, refractory to conventional therapy. It is noteworthy that patients had never been exposed to biologics in the past. The choice of treatment was influenced only by age, which was higher in the VDZ group. Therefore, although retrospective and not randomized, this study analyzed two well matched populations for major confounders.

### Previous studies comparing effectiveness of anti-TNF vs. VDZ

No prospective randomized head-to-head trial of anti-TNF vs. anti-integrin agents in IBD have been published, except for the VARSITY study^18^ in which VDZ and ADA were compared in a phase 3b, randomized, double-blind trial enrolling subjects with moderate-to-severe UC. VDZ was superior to ADA for the primary endpoint of CR at 52 weeks. Furthermore, VDZ showed higher rate of mucosal healing and a lower incidence of AEs during the treatment period. However, there were some potential biases to acknowledge. First, despite the absence of prospective randomized trial comparing IFX to ADA in UC, a network meta-analysis showed superior efficacy of IFX vs. ADA as first line biologic therapy and both the ECCO and AGA guidelines recommend IFX rather than ADA as first line therapy in UC^19,20^. Consequently, there are reasons to believe that IFX would have been a better competitor in a head-to-head trial towards VDZ^21^. Second, up to 25% of the subjects treated with ADA were anti-TNF exposed, potentially reducing the effectiveness in the ADA cohort. Additionally, no dose optimization was allowed. This may have affected the results of ADA, which needs dose adjustment more often than VDZ. Finally, steroid-free remission was not different between the two groups. Nevertheless, in our study we observed that VDZ was superior to IFX in the rate of clinical response at the end of IP, CR at the end of FU, drug persistency, need for optimization, and steroid-free remission (compared to patients who used steroids at baseline). In a recent multicenter real-life Italian study, similar results were obtained^22^. Indeed, Macaluso et al. showed that VDZ was superior to ADA and GOL in terms of steroid-free remission at week 52 (51.5%, 32.4%, and 29.4% for VDZ, ADA, and GOL respectively). Unfortunately, in this study IFX was not included in the analysis.

Several retrospective real-world studies have tested the efficacy of VDZ in inducing CR and clinical response. The VICTORY consortium published in 2018 the results of a real-life experience conducted in the US over 321 patients with active UC, 71% anti-TNFα exposed and 29% naïve to biologics. CR at 52 weeks was obtained in 51% of the subjects, and steroid-free CR in 37%. Only 28% discontinued the treatment due to loss of response, surgery, or severe AEs. On the other hand, on multi-variable analyses prior exposure to a TNFα antagonist was associated with a lower probability of achieving CR (OR 0.53, 95% CI 0.38–0.75) and endoscopic remission (OR 0.51, 95% CI 0.29–0.88)^23^. In the retrospective multicenter European pooled cohort study by Kopilov et al.^24^, VDZ was tested as first biologic in 134 anti-TNFα naïve patients with UC achieving 79.1% response and 39.5% CR at week 14. Among the 103 patients who were included in a maintenance analysis, 76.7% responded and 67% were in remission in a median follow-up of 42.5 weeks. The authors concluded that their data were consistent with those reported in naïve patients treated with anti-TNFα drugs in the literature. Furthermore, the rapidity of response to VDZ was comparable to that commonly observed with IFX, questioning one of the alleged weaknesses attributed to VDZ.

### Outcomes and clinical impact

In our study, the primary outcome of CR was achieved in a higher percentage for VDZ than IFX (PMS < 1: 75% VDZ vs. 32% IFX, p = 0.003). In this respect, the difference between the two groups may appear higher than expected. This is likely the result of the relatively small sample size, but also reflects the higher rate of drug persistency and the lower rate of drop-out due to adverse events in the group of VDZ. As consequence, VDZ requested less need to change treatment, fewer medical interventions, less utilization of healthcare resource, and a better quality of life. Moreover, higher CRP and PMS at baseline were predictive of response/remission, suggesting a better efficacy in more severe patients.

Clinical response at the end of induction was also significantly higher for VDZ than for IFX (VDZ 81% vs. IFX 54%, p = 0.014). Helwig et al.^25^ retrospectively compared the CR rate in 76 patients treated with VDZ and 57 with IFX, ADA or GOL. In the biologic-naïve sub-cohort of 22 VDZ and 40 anti-TNFα patients, rates of CR by week 26 were 50.1% for VDZ and 31.5% for anti-TNFα. Although numerically higher in patients treated with VDZ, the difference was not statistically significant. Alamneni et al., in ambidirectional cohort study over 27 subjects treated with IFX and 32 with VDZ (n = 18 and 13 naïve respectively) also found a similar response rate in patients with UC, with a better performance for VDZ in anti-TNFα exposed. However, the number of eligible patients was extremely low^26^.

Drug durability is a measure of persistent effectiveness of medical therapy in IBD, particularly important considering the chronic course of the disease, even if multiple therapeutic options are now available. Patel et al.^27^, in a real-world study, compared the efficacy of VDZ and IFX in a large biologic-naïve IBD cohort between 2000 and 2018. Drug persistency was higher for VDZ than IFX at 12 (84.5% vs. 77.5%, p = 0.006) and 24 months (77.6% vs. 64.6%, p = 0.0005) for aggregate data of UC and Crohn’s disease. However, in the subgroup analysis of UC the difference became statistically significant only at 24 months from the beginning of the maintenance phase. In the retrospective PANIC study by Ko Y et al.^28^ in 167 biologic naïve patients with UC, VDZ showed a better persistence compared with IFX at one-, two-, three- and four-year FU (HR: 1.67, 95% CI 1.27–2.18 P < 0.001). Bressler B et al.^29^ conducted a 24-months multicenter retrospective medical chart study (EVOLVE) comparing the effectiveness of IFX and VDZ in 376 and 221 UC patients respectively. The percentage of CR, clinical response, and mucosal healing were similar in the two groups, while drug persistency was higher for VDZ. Interestingly, prior exposure to azathioprine did not differ in the two cohorts. In the present study, drug persistence was expressed by Kaplan–Meier curves and compared with log-rank test. VDZ showed a greater drug durability as an estimate of uninterrupted treatment, regardless of the reasons for discontinuation (Fig. 2). Analyzing the causes of dropping out, primary non-response was significantly higher in IFX group (30% vs. 9%), while secondary loss of response was comparable in the two groups.

The steroid-free CR is of meaningful importance in the therapy of IBD due to the multiple and often subtle AEs of steroids in the long term^30^. In the largest real-world prospective observational study at 54 weeks published by GETAID (Groupe d’Etude Thérapeutique des Affections Inflammatoires du tube Digestif)^31^, the efficacy of VDZ was tested in 294 patients with moderate-to-severe IBD. More than 90% of them had failed one or two anti-TNFα agents. In 111 subjects with UC the steroid-free CR was obtained in 36% and 40.5% at 14 e 54 weeks respectively. Since in our study none of the patients was anti-TNFα exposed, the steroid-free remission with VDZ at the end of the observational period was expectedly higher than previously reported, while the result for IFX was in line with other studies (VDZ 94% vs. IFX 66%, p = 0.036).

Dose escalation is part of the treat-to-target approach and is allowed for both VDZ (shortening of the intervals) and IFX (shortening of the intervals, dose escalation or both). However, this strategy entails a direct and indirect increase in costs^32^. Dose escalation is widely used and effective for IFX, but still debated in the case of VDZ with several studies coming to different conclusions. In a phase 3b/4, prospective, open label, multinational, interventional study, 142 UC patients previously enrolled in the GEMINI LTS study and almost all in CR with VDZ every 4 weeks, received VDZ 300 mg every 8 weeks for 2 years. In the interim analysis, only 6.1% of the patients needed re-escalation to a 4-weeks regimen showing an excellent persistency^33^. Guidi et al.^34^ prospectively evaluated the role of VDZ trough levels at the end of induction in predicting drug persistency and related clinical benefit at 6, 14, 22 and 54 weeks in 59 patients with UC, 81.4% exposed to one or more anti-TNFα agents. The authors found that a VDZ trough levels cut-off of 16.55 mg/ml at week 14 was associated with drug persistency at 54 weeks and that trough levels were significantly higher in patients achieving CR at weeks 14, 22, and 54. In the study by GETAID^35^, drug optimization was used in patients with primary non-response or secondary loss of response in a 52-week follow-up. Among them, 41% were able to regain the clinical response and 30% the CR. In our study, drug optimization was decided only on a clinical basis for VDZ, and on a clinical basis plus trough levels for IFX. VDZ required less optimization than IFX and the difference was statistically significant (VDZ 28% vs. IFX 57%, p = 0.009). The delta value of 19% was likely substantial in terms of number of hospital accesses, number of infusion sessions, and inconvenience for the patients.

Safety is of utmost interest in using biologics in clinical practice and is necessarily included as an important outcome measure in clinical trials. Due to its gut selectivity, the safety profile of VDZ is generally considered superior to IFX in terms of infusion reactions, opportunistic and severe infections, especially in aged people^36,37^. In other studies, however, the safety of VDZ and anti-TNF was found equivalent^38,39^. Moreover, the role of VDZ in both inducing and mitigating flares of arthritis and sacroileitis, as well as other extraintestinal manifestations, is still under investigation^40^. In the present study, we did not observe a statistically significant difference in the occurrence of AEs between VDZ and IFX, but they were numerically higher for IFX despite the fact that the VDZ population was older. Of notice, one of the patients in the IFX group developed a reactivation of latent tuberculosis, despite a negative ELISA-QuantiFERON-TB^41^.

CRP closely correlates with the degree of systemic inflammation and is commonly used as biological marker of response. In the study by Kopilov et al.^24^ CRP level was assessed at weeks 0 and 14 in 122 UC patients treated with VDZ. Normalization was obtained in 32/72 patients (44%) with elevated CRP at baseline. We observed similar results in IFX and VDZ patients (p = 0.347; OR: 1.7; 95% CI 0.56–5.17).

## Comments

Our study has both strengths and limitations. Comparing in a real-life setting two intravenous biologic agents in a fairly homogeneous cohorts of naïve patients is of particular interest and provide additional clues to the positioning of these drugs. The majority of predetermined outcomes showed a statistically significant difference despite the small sample size, which, in turn, may have prevented from highlighting the others. Furthermore, patients in the VDZ group were enrolled over a period of 47 months restricting the potential selection bias only to the older age. In addition to the small sample size, the study has limitations inherent to all retrospective studies. CRP was not available for all patients further narrowing the sample size and potentially introducing a selection bias. Furthermore, the mucosal healing was not assessed by endoscopy. Finally, we did not perform a cost-effectiveness comparison between the two therapeutic strategies.

In conclusion, in this retrospective real-life study, VDZ was superior to IFX as a first-line therapy in biologic-naïve patients with UC for most of the predetermined endpoints (CR, persistence in therapy, clinical response at the end of induction, steroid-free remission, and need for optimization). However, the incidence of AEs and the normalization rate of CRP were similar. Further prospective and head-to-head studies are necessary to corroborate these findings and help the best positioning of current treatment in UC.

## Data Availability

The datasets used and/or analysed during the current study available from the corresponding author on reasonable request.

## Abbreviations

IFX: Infliximab
VDZ: Vedolizumab
UC: Ulcerative colitis
FU: Follow-up
CR: Clinical remission
IP: Induction phase
AEs: Adverse events
CRP: C-reactive protein
PMS: Partial Mayo score
IV: Intravenously
5-ASA: 5-Aminosalicylates
Anti-TNFα: Anti-tumor necrosis factor α
ADA: Adalimumab
GOL: Golimumab
IBD: Inflammatory bowel disease
CCS: Corticosteroids
MTX: Methotrexate
STD: Standard deviation
OR: Odds ratio
SPSS: Statistical package for social science
